# Sex Differences in Cancer-Specific Survival Are Pronounced during Adolescence and Young Adulthood: A SEER Population-Based Study

**DOI:** 10.3390/epidemiologia2030029

**Published:** 2021-09-01

**Authors:** Iyad Sultan, Justin Z. Amarin, Razan Mansour, Hala Sultan, Maysa Al-Hussaini

**Affiliations:** 1 Department of Pediatrics, King Hussein Cancer Center, Amman 11941, Jordan; iyad.y.sultan@gmail.com; 2 Office of Scientific Affairs and Research, King Hussein Cancer Center, Amman 11941, Jordan; justinzamarin@gmail.com (J.Z.A.); razanamansour@gmail.com (R.M.); 3 School of Medicine, The University of Jordan, Amman 11942, Jordan; lalasultan@hotmail.com; 4 Department of Pathology and Laboratory Medicine, King Hussein Cancer Center, Amman 11941, Jordan

**Keywords:** epidemiology, neoplasms, SEER program, sex characteristics, survival analysis

## Abstract

Sex differences in cancer survival may be related to hormonal changes during puberty and menopause; therefore, we investigated sex differences in the cancer-specific survival rates of children, adolescents and young adults (AYAs), and older adults with sex-nonspecific cancers. We interrogated the November 2019 submission of the SEER 18 database and included microscopically confirmed cases of first primary malignant tumors. We stratified the dataset into children (<15 years), AYAs (modified; 15–49 years), and older adults (≥50 years). For each age group, we used the Kaplan–Meier method to estimate the sex-stratified 5-year all-site cancer-specific survival probabilities. Of 3,386,276 eligible patients, 45,124 (1.3%) were children, 548,158 (16.2%) were AYAs, and 2,792,994 (82.5%) were older adults. The 5-year all-site cancer-specific survival probabilities were 84.0% (95% CI, 83.5%–84.5%) for boys, 84.8% (95% CI, 84.3%–85.3%) for girls, 70.4% (95% CI, 70.2%–70.6%) for male AYAs, 80.8% (95% CI, 80.6%–81.0%) for female AYAs, 52.0% (95% CI, 51.9%–52.1%) for older adult men, and 52.2% (95% CI, 52.1%–52.3%) for older adult women. The all-site survival rate for female patients with cancer is markedly higher than for male patients with cancer during adolescence and young adulthood, although this difference diminishes in older adulthood.

## 1. Introduction

The life expectancy of women is generally higher than that of men at every age. This dichotomy most likely results from differences in biology, health behaviors, and interactions between the two [1]. These differences are also important in disease and manifest in many ways, including disease presentation, prevalence, and outcomes [2]. For instance, cancer is diagnosed more frequently in men, and men are more likely to die of cancer [3].

Cook et al. performed an analysis of data from the Surveillance, Epidemiology, and End Results (SEER) Program (1973–2006) and found that the cancer-specific survival of male patients was lower for most of the 36 sex-nonspecific cancers than for female patients [4]. Micheli et al. performed a similar analysis of data from the EUROCARE-4 database and found that the relative survival of female patients was higher for most of the 26 sex-nonspecific cancers than for male patients [5]. These findings were also reproducible in population-based cohorts from other countries, including Sweden, South Korea, Estonia, and Canada [6,7,8,9].

The survival advantage of female patients with cancer is driven by many differential factors, some of which are immune-related. Some sex differences in the immune system persist throughout life, whereas others are most apparent after puberty and before menopause; therefore, both genes and sex hormones are important mediators of immunity-associated cancers [10]. Indeed, sex hormones have numerous effects on the innate and adaptive immune systems—some overlapping and others distinct. Such distinct effects on the immune system can have profound consequences on the differential health of male and female patients [11]. For example, estrogens can suppress oncogenesis but promote autoimmunity, whereas androgens can suppress autoimmunity but promote oncogenesis. Consequently, male and female patients are disproportionately affected by cancers and autoimmune diseases, respectively [11,12].

We hypothesized that puberty and menopause are important landmarks of the differential cancer susceptibility and mortality rates between the sexes; hence, age-related patterns in the sexual dimorphism of cancer-specific survival may become more apparent by stratifying patients according to these two landmarks. As such, we investigated sex differences in the cancer-specific survival of children, adolescents and young adults (AYAs), and older adults with sex-nonspecific cancers.

## 2. Materials and Methods

The SEER Program by the National Cancer Institute collects survival data from population-based cancer registries throughout the United States [13]. The SEER 18 database includes data from 18 registries: Alaska, Connecticut, Detroit, Atlanta, Greater Georgia, Rural Georgia, San Francisco–Oakland, San Jose–Monterey, Greater California, Hawaii, Iowa, Kentucky, Los Angeles, Louisiana, New Mexico, New Jersey, Seattle–Puget Sound, and Utah. SEER 18 covers approximately 27.8% of the population and contains one record for each of the 8,131,919 tumors [14].

We used SEER*Stat (version 8.3.6.1, National Cancer Institute, Bethesda, MD, United States, 2020) to access the SEER 18 database (November 2019 submission 2000–2017). We created a case listing session and included microscopically confirmed cases of the first primary malignant tumors. On the basis of the “site recode ICD-O-3/WHO 2008” variable, we excluded tumors of the breast, female genital system, and male genital system. We also excluded the ICD-O-3 morphology codes 8590–8679 (specialized gonadal neoplasms). In addition, we excluded cases with no calculated survival time or missing or unknown causes of death. For all cases, we retrieved the following SEER variables: age recode with single ages and 85+, sex, site recode ICD-O-3/WHO 2008, SEER historic stage A (1973–2015), SEER combined summary stage 2000 (2004+), survival months, and SEER cause-specific death classification. Finally, we executed the query and exported the dataset in CSV format.

We imported the data into R (version 4.0.2, R Core Team, Vienna, Austria, 2020) to perform our analyses. First, we stratified the dataset into three groups: children (<15 years), AYAs (modified; 15–49 years), and older adults (≥50 years). Next, we computed summary statistics to describe each stratum, as well as the full sample. We then plotted sex-specific survival curves for each age group by using the Kaplan–Meier method and estimated the 5-year all-site cancer-specific survival probabilities (i.e., our primary measure) and 95% confidence intervals (CIs). For each age group, we also estimated the all-site and site-specific hazard ratios (HRs) and 95% CIs of cancer-specific deaths (i.e., our secondary measure). For the site-specific analyses, we excluded any sites with less than 20 outcome events and any assorted tumors (i.e., tumors grouped under the heading “miscellaneous” or under a heading that included the word “other,” except for “other endocrine including thymus”).

## 3. Results

Our query returned 3,386,276 of 8,131,919 records (41.6%). Each record was complete and represented a unique patient, and we included all of the records in our final analyses. Of all patients, 45,124 (1.3%) were children, 548,158 (16.2%) were AYAs, and 2,792,994 (82.5%) were older adults. Overall, 1,893,388 (55.9%) patients were male and 1,492,888 (44.1%) patients were female. Of the children, 24,458 (54.2%) were boys and 20,666 (45.8%) were girls. Of the AYAs, 275,514 (50.3%) were male and 272,644 (49.7%) were female. Of the older adults, 1,593,416 (57.1%) were men and 1,199,578 (42.9%) were women. The site-specific frequencies for each age group are summarized in the Supplementary Files (see Tables S1–S3).

**Figure 1:**
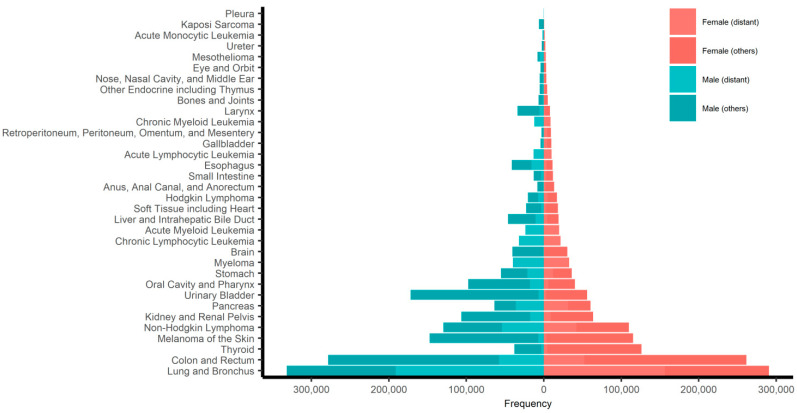
Pyramid plot of the site-specific frequencies of sex-nonspecific cancers stratified by sex. The frequencies of cancers with distant metastases (according to SEER historic stage A or SEER combined summary stage 2000) are indicated.

**Figure 2:**
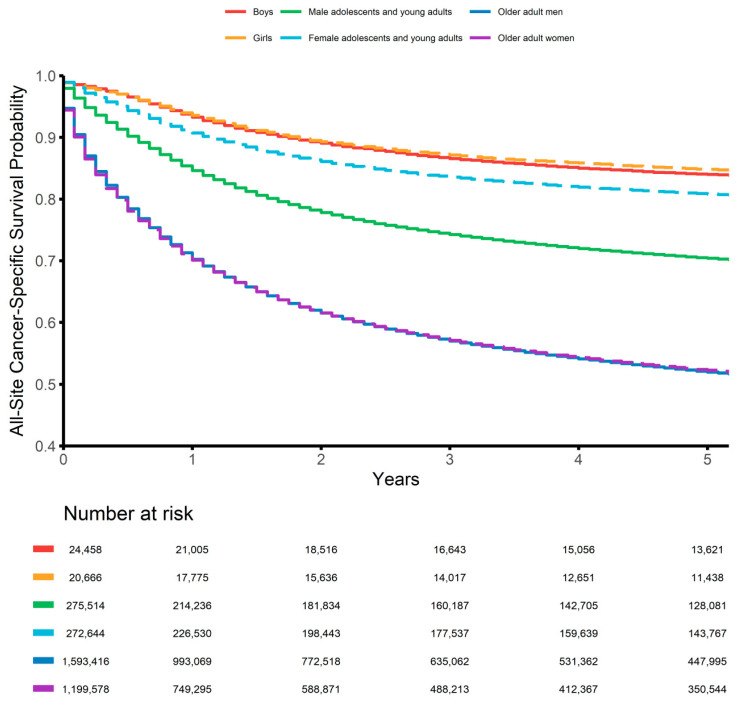
Sex-stratified Kaplan–Meier estimates of the all-site cancer-specific survival proportions of children (<15 years), adolescents and young adults (15–49 years), and older adults (≥50 years).

**Figure 3:**
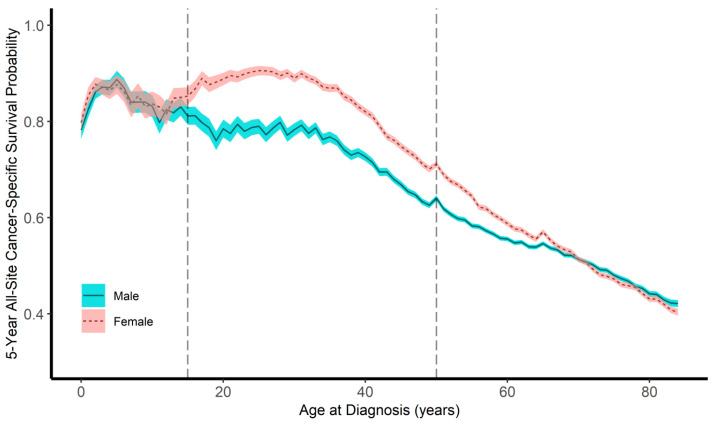
Line plot of the 5-year all-site cancer-specific survival proportion at every age (up to 84 years).

The children, AYAs, and older adults were followed for 3,788,161 person-months (median, 72 months), 40,873,715 person-months (median, 59 months), and 124,329,922 person-months (median, 22 months), respectively. During the follow-up periods, 6849 children (15.2%), 132,976 AYAs (24.3%), and 1,256,214 older adults (45.0%) died of their disease. We generated pyramid plots of the site-specific frequencies of cancers, stratified by sex (Figure 1). The 5-year all-site cancer-specific survival probabilities were 84.0% (95% CI, 83.5%–84.5%) for boys, 84.8% (95% CI, 84.3%–85.3%) for girls, 70.4% (95% CI, 70.2%–70.6%) for male AYAs, 80.8% (95% CI, 80.6%–81.0%) for female AYAs, 52.0% (95% CI, 51.9%–52.1%) for older adult men, and 52.2% (95% CI, 52.1%–52.3%) for older adult women. The all-site sex-specific survival curves for children, AYAs, and older adults are shown in Figure 2. Line plots of the 5-year all-site cancer-specific survival proportion at every age (up to 84 years) are shown in Figure 3.

**Figure 4:**
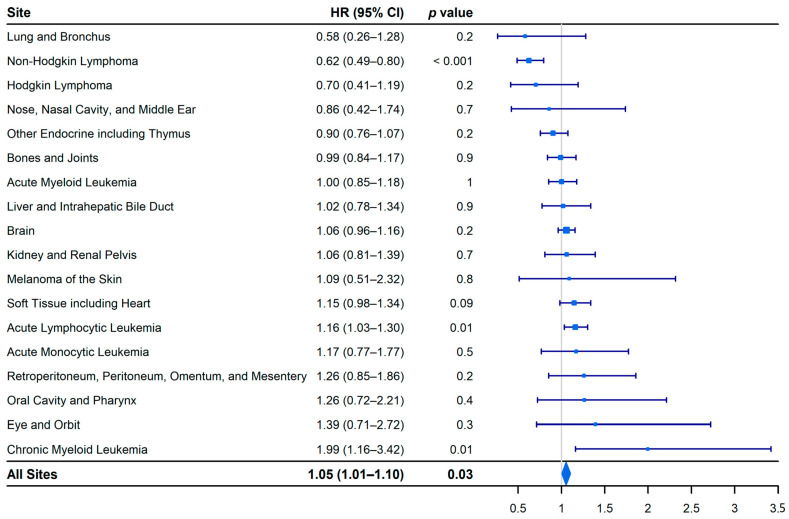
Forest plot of the site-specific hazard ratios of cancer-specific death for boys and girls (<15 years). Girls are the reference group.

The all-site hazard of cancer-specific deaths was 5% higher in boys than in girls (HR, 1.05; 95% CI, 1.01 to 1.10; *p* = 0.03; Figure 4). Boys had a statistically significant survival advantage for non-Hodgkin lymphoma (HR, 0.62; 95% CI, 0.49 to 0.80; *p* < 0.001) but a statistically significant survival disadvantage for acute lymphocytic leukemia (HR, 1.16; 95% CI, 1.03 to 1.30; *p* = 0.01) and chronic myeloid leukemia (HR, 1.99; 95% CI, 1.16 to 3.42; *p* = 0.01).

**Figure 5:**
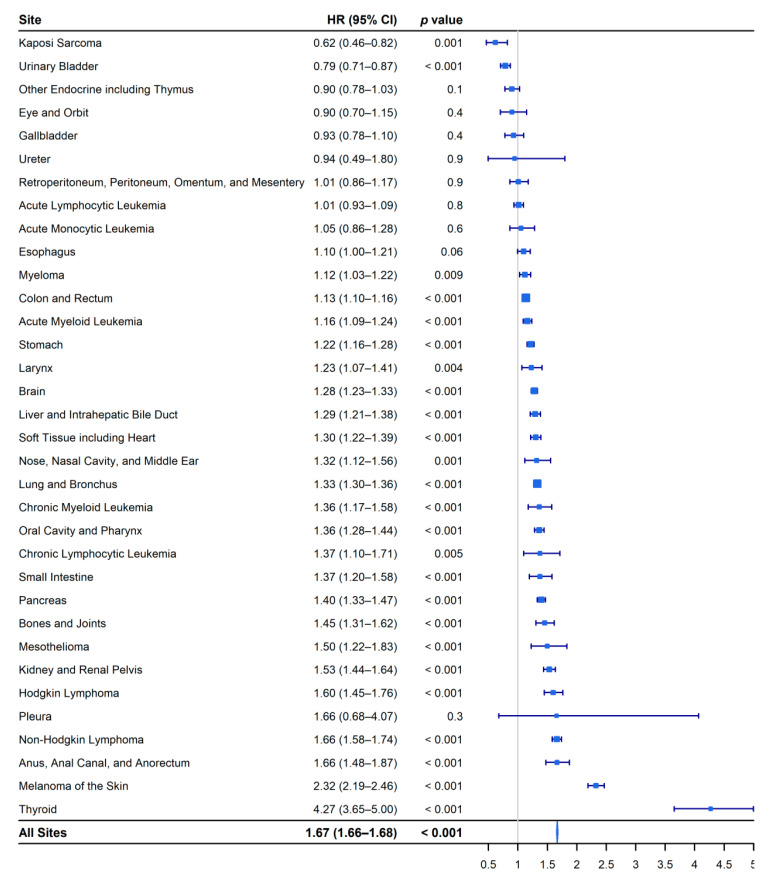
Forest plot of the site-specific hazard ratios of cancer-specific death for male and female adolescents and young adults (15–49 years). Female adolescents and young adults are the reference group.

The all-site hazard of cancer-specific death was 67% higher in male AYAs than in female AYAs (HR, 1.67; 95% CI, 1.66 to 1.68; *p* < 0.001). Female AYAs had a statistically significant survival advantage over male AYAs for 23 sites, whereas male AYAs had a statistically significant survival advantage for two sites (Figure 5).

**Figure 6:**
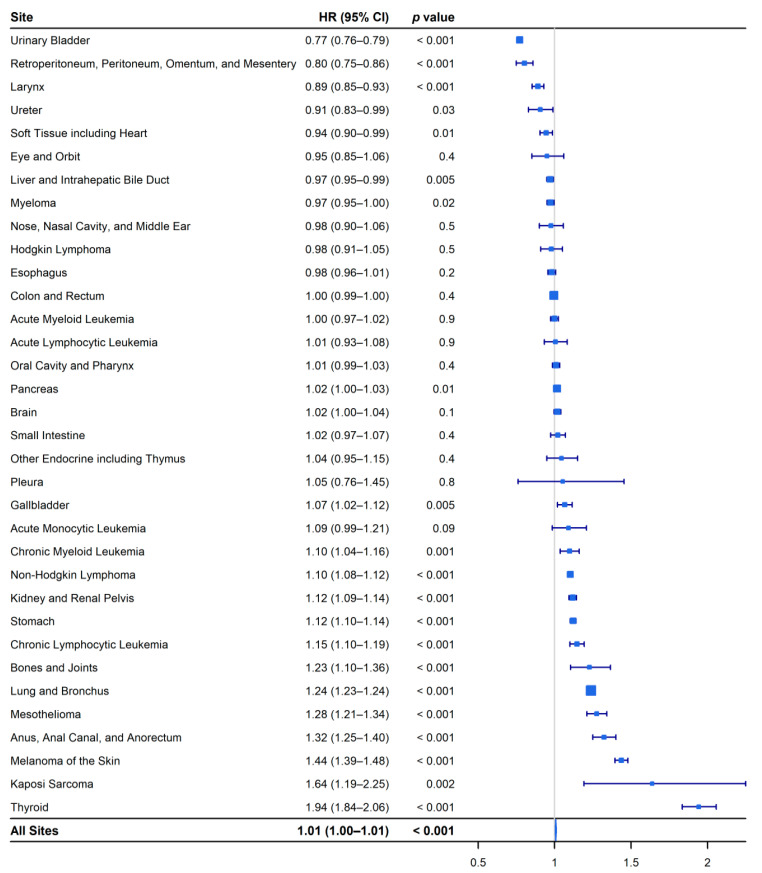
Forest plot of the site-specific hazard ratios of cancer-specific death for older adult men and women (≥50 years). Older adult women are the reference group.

The all-site hazard of cancer-specific death was 1% higher in older adult men than in older adult women (HR, 1.01; 95% CI, 1.00 to 1.01; *p* < 0.001). Older adult women had a statistically significant survival advantage over that of older adult men for 14 sites, although older adult men had a statistically significant survival advantage over older adult women for seven sites (Figure 6).

## 4. Discussion

Data regarding sex differences in cancer susceptibility and mortality rates are important because they may be used to identify health behaviors and biological targets that are amenable to interventions. We studied sex differences in the cancer-specific survival rates of children, AYAs, and older adults with sex-nonspecific cancers. We found that the 5-year all-site cancer-specific survival probabilities were comparable (<1% difference) between boys and girls and older adult men and women, although a marked difference between male and female AYAs was evident. The 5-year all-site cancer-specific survival probability for male AYAs was 10.4% lower than that for female AYAs, while the all-site hazard of cancer-specific death was 67% higher in male AYAs. We also found that girls had two favorable sites and one unfavorable site, female AYAs had 23 favorable sites and two unfavorable sites, and older adult women had 14 favorable sites and seven unfavorable sites.

Generally, across all ages, the incidence rates of most cancers are higher in male patients than in female patients [15]. In addition, the cancer-specific survival rates of male patients are lower for most cancers [4]. These findings were based on data obtained from the November 2007 submission of SEER 9 (1975–2004) and the November 2008 submission of SEER 17 (1973–2006). The results from other studies of population-based cohorts were similar [5,6,7,8,9]. To glean insights into the role sex hormones play in cancer incidence and survival differences, we stratified our analyses according to the ages of puberty and menopause, in which the levels of sex hormones in female patients are the most markedly different. In general, our results, which were based on data from the November 2019 submission of SEER 18 (2000–2017), also demonstrated the same trends as those reported in previous studies; however, we found that sex differences in the 5-year all-site cancer-specific survival probabilities and all-site hazard rates for cancer-specific death were the most pronounced in AYAs.

The 5-year all-site cancer-specific survival probabilities we report represent an aggregate measure of the 5-year site-specific cancer-specific survival probabilities weighted by the relative frequencies of the cancers in each site. On the basis of this definition, two independent factors may explain the sex differences we observed in the 5-year all-site cancer-specific survival probabilities in AYAs. First, the relative frequency of aggressive cancers may be higher in male AYAs than in female AYAs. Conversely, the relative frequency of indolent cancers may be higher in female AYAs. Second, the cancer-specific survival probabilities may be lower in male AYAs (and higher in females) for cancers of the same site. Our results show that both factors contribute to these sex differences.

Sex differences in health behaviors may account for the disparity in AYAs. In relation to cancer, health risk behaviors, early detection, psychological adaptation, and social adjustment differ between the sexes. The most notable examples of health risk behaviors are tobacco smoking and alcohol drinking. More men smoke tobacco cigarettes and abuse alcohol than do women. Together, these risk factors are related to 75% of cancers of the oral cavity and pharynx [16]. Indeed, we found that the frequency ratio for cancers of the oral cavity and pharynx was 1.0 between boys and girls, 2.2 between male and female AYAs, and 2.5 between older adult men and women. Interestingly, the corresponding HR values for death caused by cancers of the oral cavity and pharynx were 1.26 (0.72–2.21), 1.36 (1.28–1.44), and 1.01 (0.99–1.03) for children, AYAs, and older adults, respectively; therefore, the hazard of death is higher in male AYAs than in female AYAs, although these sex differences are not present in older adults. These age-related sex differences may be due to biology alone, as well as the interplay between health behaviors and biology. In addition, a working group of the International Agency for Research on Cancer identified sufficient evidence for the contribution of excess body fatness to the risk of 13 cancers, namely cancers of the gastric cardia, colorectum, liver, gallbladder, pancreas, uterine corpus, ovary, and thyroid, as well as esophageal adenocarcinoma, postmenopausal breast cancer, renal cell carcinoma, meningioma, and multiple myeloma [17]. Interestingly, the worldwide age-standardized mean body mass index is higher in women than in men, and the estimates for both sexes have been trending upward historically [18]; therefore, it is not surprising that the estimated proportion of cancers attributable to excess body fatness is twice as high in women 30 years of age or older in the United States [19]. We speculate that sex differences in excess body fatness may account for some differences in site-specific frequencies between the sexes in our study. For example, we found that the male-to-female absolute frequency ratios for cancers of the gallbladder and thyroid—both of which are obesity-related cancers—were 0.4 and 0.2, respectively; however, we could not assess sex differences in body mass index because such data are not collected by SEER.

Most biological sex differences can be attributed to the sex chromosomes. In female patients, some tumor suppressor genes on the lyonized X chromosome escape inactivation. In accordance with the Knudson two-hit hypothesis, Dunford et al. showed that the biallelic expression of these genes affords female patients more protection against cancer than that for male patients, which may in part explain the higher incidence rates of cancers in male patients. Interestingly, some genes that do not escape lyonization in healthy cells aberrantly escape lyonization in cancer cells, which may explain sex differences in cancer-specific survival [20]. In male patients, age-related loss of chromosome Y is associated with cancer, while tobacco smoking is associated with reversible mosaic loss of chromosome Y, further highlighting the interplay between health behavior and biology [21,22]. In addition to a higher risk of cancer, mosaic loss of chromosome Y portends reduced survival [23]. Sex differences that affect cancer susceptibility and mortality rates extend to DNA copy number variations, single nucleotide polymorphisms, DNA methylation, mRNA expression, microRNA expression, and protein expression [24,25,26].

In children and young adults, our site-specific analyses revealed many sex differences that were otherwise obfuscated in the all-site analysis. Interestingly, some sex differences reversed from one age group to another. For example, melanoma is more common in female AYAs than in male AYAs; however, melanoma is more common in older adult men than in women. Nevertheless, female patients have better survival probabilities than male patients in both age groups [27]. Site-specific trends are important to fully understand because they may reveal targets for intervention. Indeed, Yuan et al. studied 114 clinically actionable genes and found that 60 (>50%) showed sex-biased signatures in seven of eight cancers, suggesting that sex-specific therapeutic strategies are needed for many cancers [25].

The main limitation of our study is our choice of cutoff ages. The cutoffs we selected for the ages at puberty and menopause were based on the lower limit of the standard age range that defines AYA (according to the National Cancer Institute) and the mean age at menopause in the United States, respectively [28,29]; however, the ages of boys and girls at puberty are different, and both cutoff ages we selected were based on the average, meaning they cannot account for interindividual variability. Ideally, the groups should be constructed according to individual ages of puberty and menopause onset. Another limitation of our study is the imbalance between groups; the smallest subset included 45,124 children, whereas the largest subset included 2,792,994 older adults. This limits direct comparisons between groups because our analyses were differentially powered to detect effects of small magnitude.

## 5. Conclusions

The all-site survival advantage of female patients with cancer over that of male patients with cancer becomes pronounced during the AYA period, although this difference is mitigated in older adulthood. Site-specific trends in children, AYAs, and older adults are more diverse and warrant individual consideration. Our findings highlight the need for sex-specific primary prevention interventions and therapeutic strategies, as well as further research to explore mechanisms that underpin the pronounced survival disparity between male and female AYAs.

## Data Availability

The data that support the findings of this study are available at www.seer.cancer.gov (accessed on 31 August 2021).

## Supplementary Materials

The following are available online at https://www.mdpi.com/article/10.3390/epidemiologia2030029/s1: Table S1: Absolute and relative site-specific frequencies and site-specific absolute frequency ratios for boys and girls with sex-nonspecific cancers (*n* = 45,124). Table S2: Absolute and relative site-specific frequencies and site-specific frequency ratios for male and female adolescents and young adults with sex-nonspecific cancers (*n* = 548,158). Table S3: Absolute and relative site-specific frequencies and site-specific frequency ratios for older adult men and women with sex-nonspecific cancers (*n* = 2,792,994).
