# Supplementary material for: Sex Differences in Cancer-Specific Survival Are Pronounced during Adolescence and Young Adulthood: A SEER Population-Based Study
Source: Epidemiologia (Basel). 2021 Sep 1;2(3):391–401. doi: 10.3390/epidemiologia2030029 (PMC9620934; doi:10.3390/epidemiologia2030029)
Supplement: Supplementary file 1 [file epidemiologia-02-00029-s001.zip › epidemiologia-1269606-supplementary.pdf]

epidemiologia

Supplementary Materials: Sex Differences in Cancer-Specific Survival Are Pronounced During Adolescence and Young Adulthood: A SEER Population-Based Study

Table S1. Absolute and relative site-specific frequencies and site-specific absolute frequency ratios for boys and girls with sex-nonspecific cancers (n = 45,124).

Site	Frequencies		Absolute Frequency Ratio (M:F)
	**Boys, n = 24,458 (%)**	**Girls, n = 20,666 (%)**	
Non-Hodgkin lymphoma	2,035 (8.3)	939 (4.5)	2.2
Hodgkin lymphoma	1,090 (4.5)	689 (3.3)	1.6
Chronic myeloid leukemia	217 (0.9)	147 (0.7)	1.5
Liver and intrahepatic bile duct	578 (2.4)	393 (1.9)	1.5
Other endocrine, including thymus	1,150 (4.7)	817 (4.0)	1.4
Acute monocytic leukemia	136 (0.6)	110 (0.5)	1.2
Acute lymphocytic leukemia	6,944 (28.4)	5,726 (27.7)	1.2
Brain	4,177 (17.1)	3,522 (17.0)	1.2
Bones and joints	1,182 (4.8)	1,034 (5.0)	1.1
Acute myeloid leukemia	1,116 (4.6)	981 (4.7)	1.1
Eye and orbit	648 (2.6)	574 (2.8)	1.1
Soft tissue, including heart	1,621 (6.6)	1,585 (7.7)	1.0
Oral cavity and pharynx	219 (0.9)	222 (1.1)	1.0
Retroperitoneum, peritoneum, omentum, and mesentery	230 (0.9)	238 (1.2)	1.0
Nose, nasal cavity, and middle ear	57 (0.2)	62 (0.3)	0.9
Kidney and renal pelvis	1,230 (5.0)	1,365 (6.6)	0.9
Lung and bronchus	62 (0.3)	72 (0.3)	0.9
Melanoma of the skin	271 (1.1)	331 (1.6)	0.8

Abbreviations: F, female; M, male.

Table S2. Absolute and relative site-specific frequencies and site-specific frequency ratios for male and female adolescents and young adults with sex-nonspecific cancers (n = 548,158).

	Frequencies		
**Site**	**Male AYAs, n = 275,514 (%)**	**Female AYAs, n = 272,644 (%)**	**Absolute Frequency Ratio (M:F)**
Kaposi sarcoma	4,215 (1.5)	118 (0.0)	35.7
Esophagus	3,330 (1.2)	692 (0.3)	4.8
Larynx	3,061 (1.1)	1,003 (0.4)	3.1
Urinary bladder	9,566 (3.5)	3,287 (1.2)	2.9
Liver and intrahepatic bile duct	4,685 (1.7)	1,787 (0.7)	2.6
Oral cavity and pharynx	16,727 (6.1)	7,679 (2.8)	2.2
Chronic lymphocytic leukemia	2,133 (0.8)	1,205 (0.4)	1.8
Pleura	23 (0.0)	13 (0.0)	1.8
Kidney and renal pelvis	18,975 (6.9)	11,221 (4.1)	1.7
Acute lymphocytic leukemia	4,053 (1.5)	2,409 (0.9)	1.7
Ureter	73 (0.0)	44 (0.0)	1.7
Nose, nasal cavity, and middle ear	1,147 (0.4)	695 (0.3)	1.7
Chronic myeloid leukemia	3,671 (1.3)	2,389 (0.9)	1.5
Non-Hodgkin lymphoma	27,015 (9.8)	17,797 (6.5)	1.5
Bones and joints	3,333 (1.2)	2,319 (0.9)	1.4
Brain	12,535 (4.5)	8,778 (3.2)	1.4
Eye and orbit	755 (0.3)	562 (0.2)	1.3
Myeloma	3,876 (1.4)	2,964 (1.1)	1.3
Stomach	6,574 (2.4)	5,082 (1.9)	1.3
Other endocrine, including thymus	1,503 (0.5)	1,162 (0.4)	1.3
Soft tissue, including heart	7,350 (2.7)	5,917 (2.2)	1.2
Pancreas	5,318 (1.9)	4,406 (1.6)	1.2

Mesothelioma	357 (0.1)	302 (0.1)	1.2
Small intestine	2,093 (0.8)	1,822 (0.7)	1.1
Hodgkin lymphoma	13,027 (4.7)	11,401 (4.2)	1.1
Colon and rectum	34,590 (12.6)	30,835 (11.3)	1.1
Acute myeloid leukemia	4,983 (1.8)	4,683 (1.7)	1.1
Lung and bronchus	17,407 (6.3)	16,982 (6.2)	1.0
Anus, anal canal, and anorectum	2,155 (0.8)	2,118 (0.8)	1.0
Acute monocytic leukemia	340 (0.1)	339 (0.1)	1.0
Melanoma of the skin	31,900 (11.6)	42,469 (15.6)	0.8
Retroperitoneum, peritoneum, omentum, and mesentery	629 (0.2)	1,037 (0.4)	0.6
Gallbladder	313 (0.1)	745 (0.3)	0.4
Thyroid	16,321 (5.9)	69,754 (25.6)	0.2

Abbreviations: F, female; M, male.

Table S3. Absolute and relative site-specific frequencies and site-specific frequency ratios for older adult men and women with sex-nonspecific cancers (*n* = 2,792,994).

	**Frequencies**		
**Site**	**Older Adult Men, *n* = 1,593,416 (%)**	**Older Adult Women, *n* = 1,199,578 (%)**	**Absolute Frequency Ratio (M:F)**
Larynx	30,734 (1.9)	7,103 (0.6)	4.3
Kaposi sarcoma	2,022 (0.1)	525 (0.0)	3.9
Esophagus	37,917 (2.4)	10,698 (0.9)	3.5
Mesothelioma	7,722 (0.5)	2,407 (0.2)	3.2
Urinary bladder	162,174 (10.2)	52,798 (4.4)	3.1
Oral cavity and pharynx	80,670 (5.1)	32,452 (2.7)	2.5
Liver and intrahepatic	41,006 (2.6)	16,845 (1.4)	2.4

bile duct			
Pleura	163 (0.0)	92 (0.0)	1.8
Kidney and renal pelvis	86,018 (5.4)	51,074 (4.3)	1.7
Melanoma of the skin	115,242 (7.2)	72,494 (6.0)	1.6
Stomach	48,606 (3.1)	30,950 (2.6)	1.6
Chronic lymphocytic leukemia	29,884 (1.9)	20,563 (1.7)	1.5
Nose, nasal cavity, and middle ear	3,875 (0.2)	2,696 (0.2)	1.4
Ureter	2,603 (0.2)	1,909 (0.2)	1.4
Eye and orbit	2,809 (0.2)	2,069 (0.2)	1.4
Chronic myeloid leukemia	8,276 (0.5)	6,120 (0.5)	1.4
Brain	23,772 (1.5)	18,187 (1.5)	1.3
Hodgkin lymphoma	6,338 (0.4)	4,874 (0.4)	1.3
Acute monocytic leukemia	1,083 (0.1)	841 (0.1)	1.3
Soft tissue, including heart	13,642 (0.9)	10,847 (0.9)	1.3
Acute myeloid leukemia	17,615 (1.1)	14,088 (1.2)	1.3
Bones and joints	2,202 (0.1)	1,801 (0.2)	1.2
Myeloma	35,887 (2.3)	29,777 (2.5)	1.2
Lung and bronchus	314,317 (19.7)	273,759 (22.8)	1.1
Non-Hodgkin lymphoma	100,408 (6.3)	91,096 (7.6)	1.1
Small intestine	10,791 (0.7)	10,003 (0.8)	1.1
Colon and rectum	243,660 (15.3)	230,567 (19.2)	1.1

Acute lymphocytic leukemia	2,155 (0.1)	2,059 (0.2)	1.0
Pancreas	58,427 (3.7)	55,858 (4.7)	1.0
Other endocrine, including thymus	2,420 (0.2)	2,380 (0.2)	1.0
Anus, anal canal, and anorectum	6,193 (0.4)	11,399 (1.0)	0.5
Gallbladder	3,803 (0.2)	9,104 (0.8)	0.4
Thyroid	21,324 (1.3)	55,724 (4.6)	0.4
Retroperitoneum, peritoneum, omentum, and mesentery	1,888 (0.1)	7,924 (0.7)	0.2

Abbreviations: F, female; M, male.
